# The Effectiveness of the Gnana Laryngeal Mask Airway II (GLA-II) With Novel Suction Tubing in Gastrointestinal (GI) Cases

**DOI:** 10.7759/cureus.69103

**Published:** 2024-09-10

**Authors:** Shahab Ahmadzadeh, Landyn D Johnson, William T Barham, James Ilochi, Matthew Fredericks, Giustino Varrassi, Sahar Shekoohi, Alan D Kaye

**Affiliations:** 1 Department of Anesthesiology, Louisiana State University Health Sciences Center, Shreveport, USA; 2 School of Medicine, Louisiana State University Health Sciences Center, Shreveport, USA; 3 School of Medicine, Louisiana State University Health Sciences Center, New Orleans, USA; 4 School of Medicine, St. George’s University School of Medicine, West Indies, GRD; 5 School of Medicine, St. George’s University School of Medicine, St. George, GRD; 6 Department of Pain Medicine, Paolo Procacci Foundation, Rome, ITA

**Keywords:** gla-ii, gnana, laryngeal mask airway ii, oropharyngeal secretion, suction tubing

## Abstract

Introduction: The Gnana laryngeal mask airway II (GLA-II) is a supraglottic airway device similar to the classic laryngeal mask airway, except it has an additional suction port. This suction port allows for the removal of secretions and saliva. A previous version of the Gnana laryngeal airway 4 was made of silicone, while this newer version is made of polyvinyl chloride (PVC), which is more affordable. This study aimed to demonstrate the effectiveness and tolerability of this PVC-designed GLA-II and evaluate its ability to suction secretions.

Methods: The prospective cohort study included 100 gastrointestinal (GI) cases to determine the effectiveness and toleration of the GLA-II. The American Society of Anesthesiologists (ASA) class 1-3 patients were evaluated with a Mallampati airway score for GI-related procedures. After anesthesia induction with propofol, the GLA-II was inserted, and the time for successful insertion was recorded. All cases were completed within 62 minutes. During this time, the secretion volumes were also measured.

Results: One hundred patients were included in the study: 52% were males and 48% were females. Thirty-four patients were scored as ASA class 1 or 2, while 66 were scored as ASA class 3. The GLA-II insertion was successful on the first attempt in 92 patients, and a second attempt was necessary for six patients. It was unsuccessful in two patients. The average time for successful insertion was 28.3 ± 4.3 seconds. The average amount of saliva suctioned was 9.3 ± 2.6 mL. There were no intraoperative or postoperative complications during these cases.

Conclusion: The PVC GLA-II device is distinguished by its ability to allow suctioning during placement. With an adequate epiglottic seal, it can be safely and successfully inserted in a short period of time. More research should be conducted to explore the use of GLA-II devices in other settings, such as emergencies and life-saving scenarios.

## Introduction

The laryngeal mask airway (LMA), a form of a supraglottic airway (SGA) device pioneered in the 1980s, has emerged as an alternative to an endotracheal tube (ETT) for the management of routine and emergent airways [1,2]. In contrast to the endotracheal tube, the LMA device attaches to the respiratory tract at the level of the hypopharynx rather than traversing the vocal folds to rest in the trachea, which has been shown in clinical practice to decrease hoarseness, coughing, sore throat, as well as laryngospasm [3]. Increasingly, LMAs are utilized in both in-hospital and out-of-hospital settings, exhibiting efficacy in both routine clinical practice and in urgent situations where securing an airway is time-sensitive to allow for adequate ventilation in the clinical scenario of difficult intubation or as a bridge to endotracheal intubation [4,5] Notably, LMAs are contraindicated in patients at increased risk for regurgitation, such as in the setting of uncontrolled gastroesophageal reflux disease, nonfasting, or other high-risk patients.

In recent years, new models of LMAs have been developed. Notably, second-generation Gnana laryngeal airway 4 (GLA-4), made of silicone, and Gnana laryngeal airway II (GLA-II), made of polyvinyl chloride (PVC), were developed by AirGuard LLC, El Reno, OK. These second-generation devices incorporate a lumen that allows for the aspiration of gastric fluids, and these second-generation SGA devices have expanded indications relative to the first-generation LMAs [6]. In addition, the second-generation GLA-4 and the GLA-II have a suction tube incorporated into the dorsal part of the cuff of the laryngeal mask to remove any liquids in the hypopharynx. The first-generation SGA, the GLA, made of silicone, was developed by an Australian company (Gnana Medical Australia Pvt. Limited, Gnana Medical, Australia). Following the initial description of the first-generation GLA in the Turkish Journal of Anaesthesiology and Reanimation, the authors evaluated the second-generation GLA-4 made of silicone in terms of success in time to insertion, ease of insertion, and number of required attempts in 50 patients undergoing elective endoscopy [7,8]. This second clinical investigation was published in *Cureus* in 2023. The study found that it took an average of 27.1 seconds to successfully insert, with a standard deviation of ±3.9 seconds. The tube was correctly placed in 49 out of 50 patients, and none of the 50 patients experienced hoarseness, sore throat, difficulty swallowing, or cough immediately after the operation. Additionally, the incorporation of a suction tube in the dorsal aspect of the cuff of the laryngeal mask GLA-4 allowed for intraoperative oropharyngeal secretion suction, which may potentially decrease the risk for laryngospasm and benefit patients with medical conditions such as upper airway cough syndrome or asthma [9,10].

Therefore, the current study aims to expand the evaluation of the second-generation GLA-II, which also has a suction tube incorporated on the dorsal aspect of the cuff of the laryngeal mask but is made of PVC, in a larger cohort of 100 colonoscopy cases at Louisiana State University Health Science Center at Shreveport.

## Materials and methods

**Figure 1:**
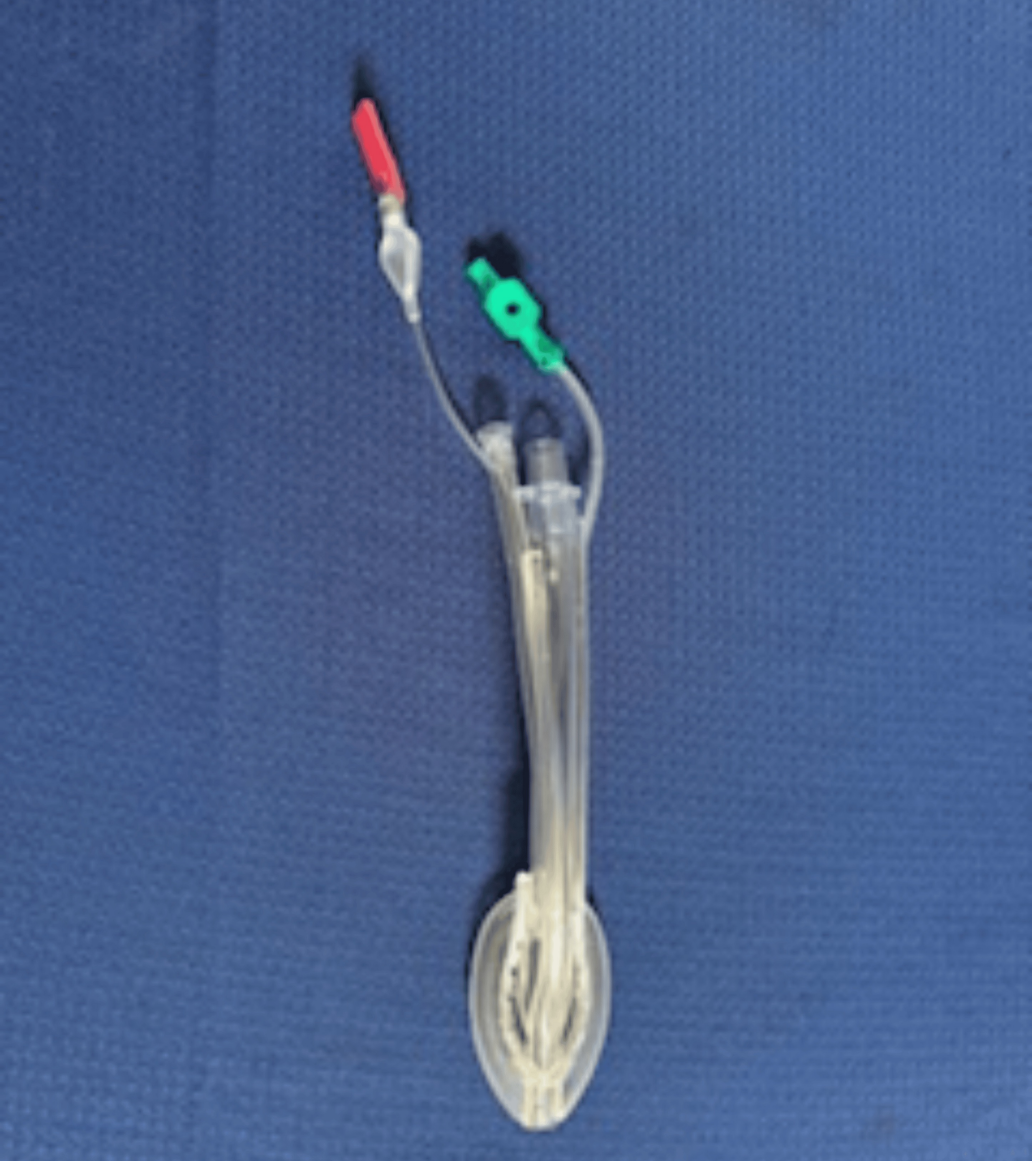
Gnana laryngeal mask airway II

The study occurred at Ochsner Louisiana State University Health Shreveport-Academic Medical Center. The facility is a level 1 trauma center in Shreveport, Louisiana. The study was approved for continuation (approval number: 00000178) by the Institutional Review Board of Louisiana State University Health Sciences Center in Shreveport. This prospective observational study included cases of patients undergoing elective colonoscopy. The primary purpose was to determine the toleration and effectiveness of the GLA-II (Figure 1) by measuring the successful insertion rate in a larger sample size of 100 patients. Other outcomes recorded were the time taken for successful insertion, the number of attempts for successful insertion, and the amount of salivary secretions suctioned. The amount of saliva removed was noted as related to the fact that the GLA-II is distinguished by its suction tube, which can prevent laryngospasms. Finally, any complications, both intraoperatively and postoperatively, were recorded during the procedures.

Patients included in this study were of either sex, scheduled for elective surgery (colonoscopy), and had a BMI less than 35 kg/m^2^. Patients excluded from this study were those with emergency procedures, difficult airways, upper respiratory tract infections, and any cardiorespiratory or cerebrovascular diseases. All patients who participated in this research study were asked to sign a consent form explaining the use of the GLA-II device as the mechanism for maintaining an airway during their elective procedures. Before the procedure, the 100 patients who participated were each assigned a class from the American Society of Anesthesiologists (ASA) classes. The ASA classes range from I to VI. Class I describes the healthiest individual, while class VI describes a brain-dead individual [11]. Only patients with a score of I, II, or III were allowed in the study. The patients were also given a Mallampati score before the procedure to report and describe any difficult airways.

Before the procedure, each patient was given an intravenous line. Basic patient monitoring was implemented with a continuous electrocardiogram, oxygen saturation, end-tidal carbon dioxide (EtCO_2_), blood pressure, heart rate, and respiratory mechanics parameters. The LMA cuff was checked for inflation and deflation before use. The mask was also lubed before placement in the larynx. General anesthesia was induced with propofol. After completing induction, the GLA-II device was placed to seal the airway.

The GLA-II device was passed to the posterior pharyngeal wall using the index finger, and then, the mask was inflated. The amount of time taken for placement of the GLA-II device was recorded. Auscultation occurred for equal and bilateral breath sounds over both lung fields to confirm that the device was correctly placed. This was also confirmed by viewing EtCO_2_ on the monitor and the absence of auscultatory air leak around the GLA-II. Three attempts at placing the device were allowed for each patient, and each patient's total number of attempts was recorded. A different size GLA-II was recommended for each patient based on the manufacturer's instructions. These instructions suggest a specific size for different weights. If the device was not working properly, the insertion depth was first increased. Next, the device was rotated and then withdrawn slightly. Maintenance of anesthesia was kept with propofol until the procedure was finished. Once the patient was awake and breathing spontaneously, the GLA-II was removed.

Statistical analysis was carried out using Microsoft Excel version 16.87 (Microsoft Corporation, Redmond, WA). All quantitative variables, including the mean and standard deviation values, were determined using measures of central tendency and dispersion.

## Results

One hundred cases, 52 males and 48 females, were chosen for this study. Of the 100 patients, 34 were ASA class II, and 66 were ASA class III. The Mallampati scores ranged from I to III for all 100 patients. Thirty-one received a Mallampati score of I, 54 received a score of II, and 15 received a score of III.

The average length of the colonoscopies was 37 minutes, with cases ranging from 15 to 62 minutes. The GLA-II insertion was successful on the first attempt in 94 patients. A second attempt was necessary for successful insertion in four patients. The first and second attempts were unsuccessful in only two patients. Therefore, successful insertion was established in 98 out of the 100 patients enrolled. The average time for successful placement was 28.3 ± 4.3 seconds (Figure 1).

**Figure 2:**
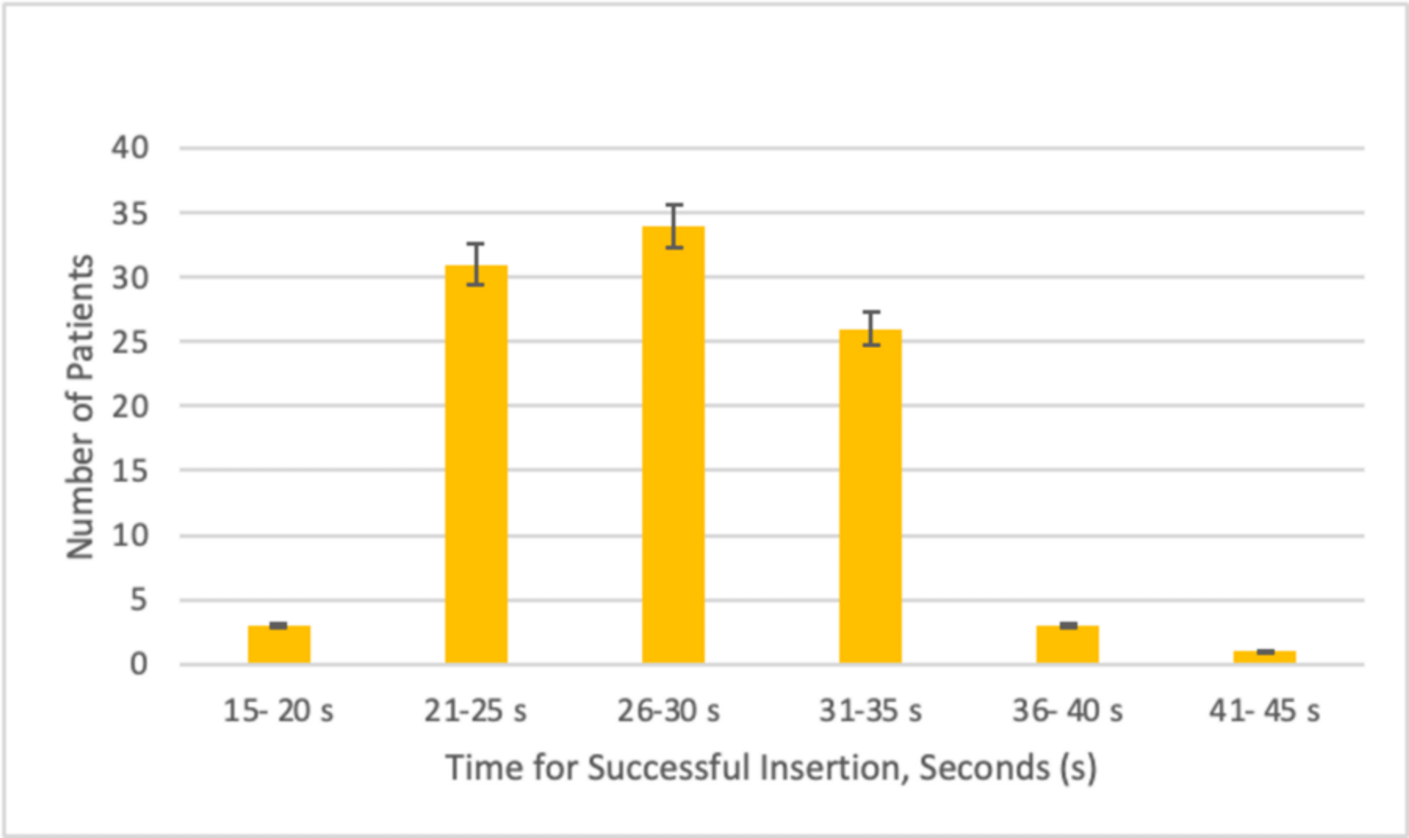
A bar graph demonstrating the time (in seconds) taken to insert the GLA-II GLA-II: Gnana laryngeal mask airway II

The total amount of saliva and secretions was measured in milliliters from the time the GLA-II device was placed until it was removed. The average amount of saliva suctioned was 9.3 ± 2.6 mL (Figure 2). No intraoperative or postoperative complications were noted in any of the 100 patients in the study using the GLA-II device. Each patient was asked questions regarding postsurgery symptoms, such as cough, hoarseness, and sore throat. No patients reported these symptoms regarding using this novel SGA device.

**Figure 3:**
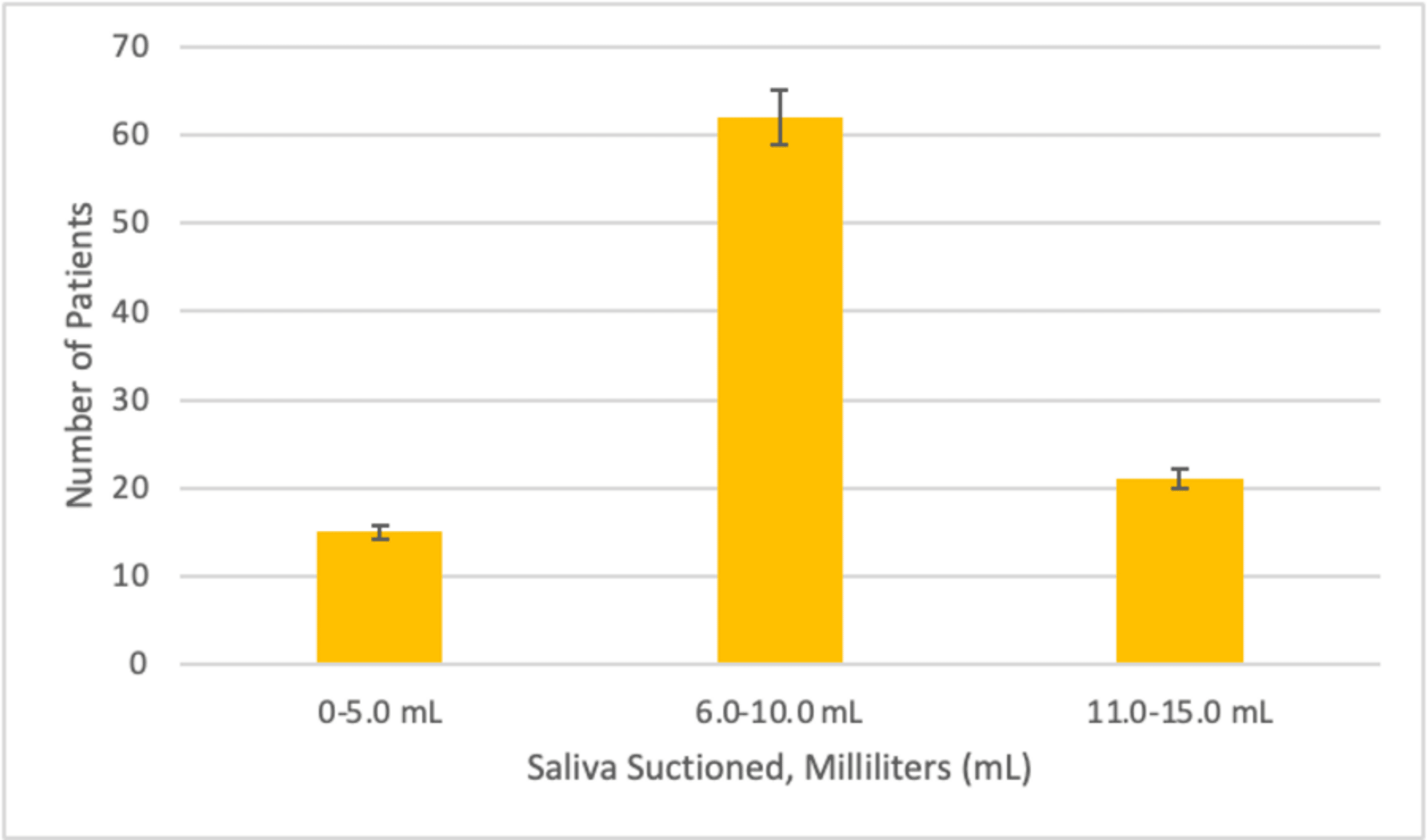
A bar graph demonstrating the amount of secretions suctioned

## Discussion

Like any device, SGAs have both advantages and disadvantages. Determining if SGAs, such as the GLA-II, are more advantageous than endotracheal intubation is crucial because of the potential clinical benefits of its technology and its application in clinical practice. In this regard, the most well-known advantages of the SGAs are a lower risk of sore throat and reduced occurrence of laryngospasms [12,13]. SGAs are also able to reverse bronchospasms [14]. SGAs are also useful in emergency airway situations when intubation is unsuccessful in assisting ventilation, but they are not recommended for patients with an increased risk of regurgitation [15]. The GLA-II is unique compared to other SGAs because it allows for suctioning oropharyngeal secretions while in place [8]. This function makes the GLA-II more beneficial than most SGAs, specifically in patients with sinusitis, secretions, and postnasal drip [16]. The continuous development of SGA models has allowed for increased use in patients and cases deemed problematic. The most common example would be the use of SGAs in obese patients. One recent study showed an increase in airway events in obese patients using SGAs; however, it also did not show a decrease in airway events in obese patients using tracheal intubation [10].

It should be noted that the SGA device has some disadvantages. The most common problems with SGAs are the potential loss of airway, displacement after insertion, and an increased risk of regurgitation [7]. Another known issue is the trauma associated with the placement of SGA devices. Major trauma has been described as damage to the tongue, uvula, and epiglottis. A too-large SGA can cause oropharyngeal nerve injury, including recurrent laryngeal nerve, resulting in vocal cord paralysis. There have also been reported injuries to other nerves, such as the lingual nerve and the hypoglossal nerve, with varying levels of morbidity. It is believed that this nerve damage is directly related to compression by the cuff, which serves to seal the laryngeal inlet following insertion of the SGAs [16,17]. It should be noted that most trauma at these locations results in bruising. Greater injuries can often be related to incorrect or forceful insertion and/or using an SGA device that is too large. The results from the previous study with a smaller cohort of 50 patients corresponded significantly to our results. The previous study produced an average insertion time of 27.1 seconds, while this study had an average insertion time of 28.3 seconds [8]. The amount of saliva suctioned per patient in both studies was also very similar, with just a 0.7 mL difference in averages. These similarities help to solidify the significance and efficacy of the GLA-II device. Another similar study focused mainly on the quantity of secretions and saliva removed from LMA devices [18]. The study showed that removing LMA deflated cuffs removed approximately 2.5 mL of saliva while removing inflated cuffs removed approximately 3 mL. Conversely, our study showed that using the GLA-II device's suction removed an average of 9.3 mL of secretions and saliva. This is nearly three times the average amount recorded in the earlier study. This difference reveals the notability of the suction on the GLA-II compared to other LMA devices.

The main limitation to this research study was the exclusion of certain patient populations that are considered high risk. These populations consisted of patients with difficult airways, cardiorespiratory or cerebrovascular disorders, class III obesity, and pregnant women. Consequently, the results established in this study cannot be applied to these high-risk patient populations, and future evaluation may better provide a clinical best practice strategy for this SGA device.

## Conclusions

This study supports the efficacy of the GLA-II, which features an innovative suction tube that can be used to substitute conventional airway devices for elective colonoscopy. Showing a very high success rate in the insertion of 98% within the first two attempts, this technique also has an impressive average time of 28.3 seconds (±4.3 seconds). The GLA-II appears to possess high safety, low side effects, and provide high patient quality and clinical effectiveness. An integrated suction tube reduces the probability of laryngospasm by managing oropharyngeal secretions. Additionally, our study did not report any patients experiencing coughing, hoarseness, or sore throat, which are often prevalent complications that occur after intubation. Such factors make the GLA-II better and more favorable than other standard and cumbersome procedures, such as endotracheal intubation, usually followed by some discomfort for the patient and a more extended recovery period.
